# Ultra-Processed Food and Prostate Cancer Risk: A Systemic Review and Meta-Analysis

**DOI:** 10.3390/cancers16233953

**Published:** 2024-11-26

**Authors:** Cayla Fichtel-Epstein, Janice Huang, Benjamin James Rich, Crystal Seldon Taswell, Derek Isrow, William Jin

**Affiliations:** 1 Miller School of Medicine, University of Miami, Miami, FL 33136, USA; 2 Department of Radiation Oncology, Sylvester Comprehensive Cancer Center, University of Miami, Miami, FL 33136, USAcstaswell@miami.edu (C.S.T.); disrow@med.miami.edu (D.I.); willjin@miami.edu (W.J.)

**Keywords:** prostate cancer, ultra-processed food, dietary pattern, chronic inflammation

## Abstract

**Background/Objectives:** Prostate cancer is the second leading cause of cancer death among American men, following lung cancer. While diet and exercise have been extensively studied in relation to prostate cancer prevention, the evidence remains inconclusive. **Methods**: A comprehensive literature search was performed to identify observational studies investigating the association between ultra-processed food (UPF) consumption and prostate cancer risk and mortality, determined by the NOVA classification system. In addition, we conducted subgroup analyses to assess the association based on study design, age, and data collection methods. **Results**: Six studies were identified, including four cohort studies and two case–control studies. No significant association was found between high UPF consumption and increased risk of prostate cancer [RR = 1.02, 95% confidence interval (CI) = 0.96–1.08, n = 5]. However, there was a slight increase in mortality (RR = 1.15, 95% CI = 0.99–1.35, n = 2). A subgroup analysis by the dietary assessment method revealed an RR of 1.01 (95% CI = 0.93–1.09) for studies using the food frequency questionnaire (FFQ) and 1.04 (95% CI = 0.93–1.16) for studies using 24-h recalls. There was no significant heterogeneity among the studies (I^2^ = 0, *p* = 0.82). **Conclusions**: This meta-analysis suggests no significant association between high UPF consumption and prostate cancer risk. Given the known associations with other chronic diseases, the potential public health implications of reducing UPF consumption remain important. Further research with the use of more robust food assignment systems and more precise dietary assessments is needed to clarify the role of UPF in prostate cancer development.

## Simple Summary

Prostate cancer is the second leading cause of cancer-related deaths among men in the United States. While genetic and environmental factors have been extensively studied, the role of modifiable lifestyle factors, particularly diet, is less understood. Ultra-processed foods, known for their high content of additives and preservatives and low levels of whole-food ingredients, have been associated with various cancers. This study aimed to determine whether ultra-processed food consumption is linked to increased prostate cancer incidence or mortality. After reviewing data from six studies, we found no significant association between ultra-processed food intake and prostate cancer risk. However, higher consumption was correlated with a slight increase in mortality. Further research is needed to clarify these associations and improve dietary assessment methods in future studies.

## 1. Introduction

Prostate cancer is the second most frequent cancer among men worldwide, with an estimated 1.4 million new cases and 375,000 deaths [1]. Australia/New Zealand, Northern American, Western and Northern Europe, and the Caribbean were found to have the highest estimated incidence rates, while the lowest rates were observed in South–Central Asia [2]. Established risk factors for prostate cancer are age, race/ethnicity, family history, and genetic predisposition, which are non-modifiable [3,4]. Modifiable risk factors such as diet, exercise, obesity, and smoking are implicated [5,6,7].

The Western diet pattern is characterized by a high intake of highly processed foods, high-sugar drinks, and high-fat products [8]. Currently, “ultra-processing”, which serves to create attractive, cheap, and hyper-palatable food, has become the dominant form of food processing in both high- and middle-income countries [9]. The role of ultra-processed food (UPF) in health was first highlighted by Monterio et al. in 2009, who later developed the NOVA food classification system [10,11]. The NOVA classification groups foods according to the extent and purpose of industrial processing [12]. There are four categories of food and food products, ranging from unprocessed foods/minimally processed foods (NOVA group 1) altered by the removal of inedible/unwanted parts to ultra-processed foods (NOVA group 4), which includes industrial formulations made with ingredients not typically used in culinary preparations for the purpose of imitating the sensorial qualities of group 1 foods [13].

The emerging evidence linking ultra-processed foods (UPFs) to an increased risk of cancer has garnered growing interest in recent years. Large-scale studies, such as the NutriNet-Santè cohort, have demonstrated that even a 10% increase in the proportion of UPFs in the diet is associated with a 12% higher risk of overall cancer [14]. Additionally, several studies have identified specific associations between UPF consumption and cancers like colorectal, breast, gastric, and ovarian cancers [15,16,17,18]. However, research focused on the relationship between UPF intake and prostate cancer remains limited.

A previous meta-analysis revealed a positive association between UPF intake and various cancers, including colorectal, breast, pancreatic, chronic lymphocytic leukemia, and central nervous system tumors. Despite this, no evidence was found linking UPF consumption to prostate cancer, likely due to the small number of studies investigating this association (*n* = 3) [19]. This highlights the need for more extensive research to better understand the potential relationship between UPF intake and prostate cancer risk.

In response to this gap in the literature, we conducted an up-to-date systematic review and meta-analysis to explore the connection between UPF consumption and prostate cancer, with a focus on prostate cancer incidence and mortality. Additionally, this review aims to describe the potential effects of UPF consumption on prostate cancer development and provide insights for future research directions.

## 2. Materials and Methods

### 2.1. Search Strategy

This study was carried out in accordance with Preferred Reporting Items for Systematic Reviews and Meta-Analyses (PRISMA). A comprehensive literature search was conducted between June–July 2024 to identify relevant studies on ultra-processed foods and prostate cancer. PubMed and Embase were searched. The search strategy combined keywords related to prostate cancer and ultra-processed foods, including (“ultraprocess*” OR “ultra process*” OR “UPF”) AND (“neoplasm” OR “tumor*” OR “cancer*” OR “malignant*” OR “carcinoma”) AND (“prostate” OR “prostatic”). The search was limited to articles published from 2009 to 2024, as the term “ultra-processed food” was introduced in 2009 [10]. Additionally, reference lists of included studies and relevant reviews were manually searched to identify any additional studies. This review was performed in accordance with the PRISMA (Preferred Reporting Items for Systematic Reviews and Meta-Analyses) guidelines and has not been registered.

### 2.2. Inclusion and Exclusion Criteria

Studies were included in this analysis if they met the following criteria: (1) prospective study (e.g., cohort, epidemiological, or cross-sectional) or retrospective studies (e.g., case-control studies); (2) subjects >18 years old; (3) exposure of UPF consumption defined by the NOVA classification system; (4) incidence or mortality outcomes of prostate cancer; (5) published in the English language.

Reviews, reports, abstracts without full text, and case reports were excluded. Animal studies and articles that did not specifically report on UPF and prostate cancer were also excluded.

### 2.3. Data Extraction and Study Quality Assessment

Two reviewers (C.E. and J.H.) independently conducted data extraction. The first author, year of publication, journal, study design, population, mean age (≥55 or <55), dietary assessment methods, risk estimates (OR or HR), and key outcomes were recorded.

The quality of each study was independently assessed by two reviewers using the Newcastle–Ottawa Scale (NOS). Scores ranged from 0–9 related to study selection, comparability of participants, and assessment of outcome/exposure. Studies with NOS scores ≥7 were considered high quality. Any disagreements were resolved through discussion or by consulting a third reviewer (W.J.).

### 2.4. Statistical Analysis

In this study, pooled relative risk was calculated with risk ratios and their corresponding 95% confidence intervals. The between-study heterogeneity was examined with Cochran’s Q test and I-square (I^2^) statistics. I^2^ values of 25%, 50%, and 75% were classified as low, moderate, and high degrees of heterogeneity, respectively. A fixed model was used with I^2^ values <25%. Otherwise, a random effect model was used. Publication bias was assessed through visual inspection of funnel plots [20]. All statistical analyses were conducted with the software R, version 4.4.1, using the package ‘meta’ [21].

## 3. Results

### 3.1. Literature Search Results

**Figure 1:**
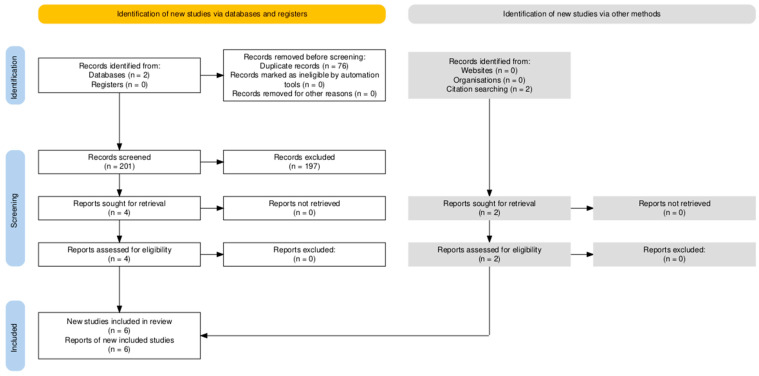
PRISMA flowchart showing the studies’ selection.

Figure 1 illustrates the PRISMA flow chart of the literature search process [22]. The PubMed and Embase search yielded 277 records. After removing duplicates, 201 studies remained for evaluation. Additionally, 197 irrelevant articles were excluded based on their titles and abstracts: these articles either did not use UPF as their exposure or did not have prostate cancer risk or mortality as their outcome. Two relevant articles were identified by reviewing references of published articles and included for analysis. In total, six articles were included in the quality evaluation. Results of the quality assessment are shown in Supplemental Table S1. All included studies had an NOS of ≥7.

### 3.2. Characteristics of the Included Studies

Four cohort studies and two case–control studies, accounting for 779,365 participants and 18,036 prostate cancer cases, were included in this systemic review and meta-analysis [14,16,23,24,25,26]. Descriptive data of included studies are shown in Table 1. All studies were published between 2009 and 2023. Included cohorts came from Europe or North America, with follow-ups ranging from 5–14.1 years. For UPF consumption assessment, two studies collected data with 24-h dietary recalls, and four studies used a food frequency questionnaire (FFQ).

### 3.3. Ultra-Processed Food Intake and Prostate Cancer

**Figure 2:**
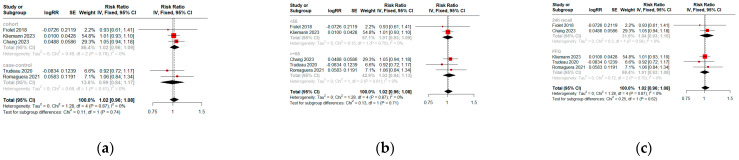
Forest plot represents the relationship between ultra-processed consumption and the risk of developing prostate cancer [14,16,23,25,26]. (**a**) Across cohort studies and case–control studies. (**b**) Stratified by age. (**c**) Stratified by dietary assessment method.

**Figure 3:**
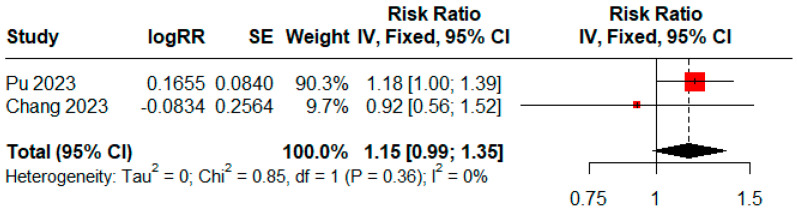
Forest plot corresponding to consumption of ultra-processed food and prostate cancer mortality [23,24].

The pooled analysis of observational studies revealed a relative risk of 1.02 (95% CI = 0.96–1.08, n = 5) for prostate cancer among individuals with high UPF consumption (Figure 2a). Two studies [23,24] identified a non-significant trend towards higher mortality risk with an RR of 1.15 (95% CI = 0.99–1.35, Figure 3). There was no significant heterogeneity among all included studies (I^2^ = 0, *p* = 0.82).

A priori subgroup analysis was also performed. When stratified by study design, pooled cohort studies have a prostate cancer risk of RR of 1.02 (95% CI = 0.96–1.09), while case–control studies had an RR of 0.99 (95% CI = 0.84–1.17, Figure 2a). When stratified by mean age, the RR is 1.01 and 1.03 for groups below or above fifty-five, respectively, suggesting that age does not significantly modify the effect of high UPF consumption on prostate cancer risk (Figure 2b). When evaluating the dietary assessment method (Figure 2c), the RR is slightly higher for studies using 24-h recall (RR = 1.04) compared to FFQ (RR = 1.01), but this difference was not significant. Table 2 summarizes the key findings and analytical methods of each study.

## 4. Discussion

Recent literature has shown associations between ultra-processed food (UPF) consumption and chronic diseases in developed countries [19,27,28,29]. Despite prostate cancer being the second leading cause of cancer death in males, there is limited evidence investigating the relationship between UPF consumption and its pathogenesis. In this systematic review and meta-analysis, we examined the association between ultra-processed food consumption and prostate cancer, focusing on prostate cancer incidence and mortality. Our findings suggest that there is no significant association between higher UPF consumption and risk of prostate cancer (RR = 1.02, 95% CI = 0.96–1.05) using current methodologies. However, it is worth noting that obesity and smoking, both of which are associated with increased prostate cancer risk or mortality [7,30,31,32], could confound these associations. Obesity, often linked to high UPF consumption due to its calorie-dense and hyper-palatable nature, is a known risk factor for high-grade and advanced prostate cancer [33]. Smoking is also associated with an increased risk of fatal prostate cancers [7]. All studies in our review accounted for BMI and smoking status. There was no significant heterogeneity among the included studies, indicating consistent findings across different populations and study designs. Additionally, our sub-group analysis revealed that age does not modify UPF-associated prostate cancer risk.

In many other disease sites, malignancies have been linked to UPF consumption. While the exact mechanisms by which UPFs contribute to carcinogenesis are not fully understood, several hypotheses have been proposed. These include exposure to non-nutritional carcinogenic food additives that act through inflammatory mediators, the use of carcinogenic processes to manufacture these foods, hormone-mediated mechanisms of endocrine disruption, and obesity-related changes resulting in higher visceral fat pad mass, systemic inflammation, and endocrine disruption [34,35,36].

Diets that produce chronic, low-grade inflammation that leads to cancer may have the most support in the literature [37]. Cohort studies have reported associations between elevated C-reactive protein concentration (CRP), an inflammatory marker, and UPF consumption [38,39,40]. Specifically, in the Melbourne cohort study, including 1261 men and 757 women, the association between the high-sensitivity C-reactive protein concentration (hsCRP) and UPF intake in 100 g increments remained robust in men, only after further adjustment of their BMI [38]. Moreover, prostate cancer risk has been separately linked with chronic low-grade inflammation [40,41,42] and metabolic syndrome [43,44], suggesting a mechanism where diet contributes to cancer risk. One such pathway is through the overconsumption of hyperpalatable micro- and macronutrients like trans fats. Higher consumption of UPF is associated with visceral fat accumulation, which contributes to systemic inflammation [44,45,46]. UPFs are often calorie-dense and highly correlated with trans fatty acid composition [47,48]. Trans fatty acids (TFAs) are unsaturated fatty acids with one or more unconjugated double bonds, and industrial trans fatty acid intake promotes inflammation and oxidative stress [49]. Michels et al. reported a significant positive association of total trans-fat with the development of prostate cancer (OR 1.49; 95% CI 1.13–1.95) [50]. Additionally, Minas et al. analyzed 24 circulating fatty acids in 2934 men, including 1431 prostate cancer cases and 1503 controls. Interestingly, trans fats are associated with increased odds of prostate cancer in distinct populations of Ghanaian, African American, and European American men, suggesting trans fatty acids as a potential risk factor independent of ancestry [51].

Furthermore, another potential mechanism by which UPFs may elevate prostate cancer risk is through the consumption of food additives and contaminants. These additives are used to enhance the flavor, texture, and color of food products, extend their shelf life, and ensure the uniform mixing of ingredients [52]. A key example is nitrates and nitrites, commonly added to processed meats to maintain color and prevent bacterial growth. While nitrates and nitrites themselves are not inherently carcinogenic, they can be converted into nitrosamines, harmful compounds that can form during food processing or within the body. Nitrosamines are known to induce oxidative stress, cause chronic inflammation, and inflict DNA damage, i.e., processes that can collectively contribute to cancer development [53].

In the French NutriNet-Santé cohort study, a high intake of nitrites, particularly sodium nitrite, was associated with an increased risk of prostate cancer. A prospective cohort study in the United States corroborated these findings, indicating that nitrites and nitrates raise the risk of advanced prostate cancer, though no significant link was found with fatal prostate cancer [54].

In addition to dietary patterns, trace elements have emerged as factors that may influence prostate cancer outcomes. Some studies have shown that specific trace element levels can affect survival rates in prostate cancer patients, underscoring the role of micronutrients in cancer progression [55]. This highlights the potential for a broader examination of dietary components beyond UPFs to include essential trace elements that may modulate prostate cancer prognosis.

The “hormonal hypothesis”, which states that androgens and estrogens play a critical role in the development and progression of prostate cancer, remains one of the most prominent theories in understanding the disease’s etiology [56]. In this context, ultra-processed foods (UPFs) have emerged as a potential concern due to the presence of endocrine disruptors (EDs) in their packaging or within the food itself. These chemical substances can mimic or interfere with natural hormonal signaling, even at very low doses [57]. It has been hypothesized that exposure to endocrine-disrupting chemicals could be one of many mechanisms linking UPF consumption to negative health outcomes. However, to date, no studies have directly investigated whether UPF intake correlates with elevated biomarkers of common EDs [58]. Nevertheless, several in vitro studies using human prostate epithelial cells and in vivo studies in animal models suggest a connection between ED exposure and an increased risk of prostate cancer (need a source).

Our study did not identify differences between the two main methodologies used for dietary assessment: 24-h dietary recall and FFQ, which are both subjective assessments. Although 24-h recalls are typically open-ended and interviewer-administered, some studies, such as NutriNet-Santé and the UK Biobank, employed web-based 24-h recalls, in which food choices are pre-defined, similar to FFQ. FFQ often has pre-defined questions that are self-administered or administered by an interviewer [59]. Both methods are prone to several limitations, including recall bias, inadequate capture of ground truth dietary intake, and insufficient representation of dietary intake. Furthermore, one has to assume that a snapshot generated by these subjective questionnaires is truly representative of dietary patterns over decades and may be one such reason that effect sizes for dietary impact on prostate cancer incidence is relatively small. Overall, accurate quantification of long-term dietary intake, as an exposure to oncologic outcomes, has been a challenge. While combining FFQ with dietary records or biomarkers may help obtain more accurate estimates [60], more robust methods of dietary assessment are needed.

Moreover, the robustness of food classification is another area receiving significant research attention. Most classification systems, including the commonly used NOVA system, lack quantitative measures and instead correlate the level of processing with nutrition [61,62]. For oncologic relevance, NOVA classifications may need further specification for carcinogenic impact. The descriptive nature can lead to variations and ambiguity. For example, in a study conducted by Braesco et al., food and nutrition specialists were surveyed to assign foods to NOVA groups [63]. The results showed low overall consistency among evaluators, highlighting the potential limitations of the current NOVA criteria.

A limitation of this review is its reliance on overall prostate cancer as the outcome, without distinguishing between low- and high-grade disease. Many studies relied on data from hospital medical records, health insurance databases, national cancer registries, and pathology reports, which can introduce detection bias [64]. Men who are more health-conscious may utilize healthcare services earlier, resulting in a diagnosis of lower-grade tumors. Conversely, those who delay seeking medical attention may only be diagnosed at more advanced stages, thus presenting with more aggressive forms of the disease [65]. Therefore, future directions should consider looking into the associations between UPFs and high-grade prostate cancer.

## 5. Conclusions

In conclusion, our meta-analysis suggests no significant association between high UPF consumption and prostate cancer risk. However, given the established links between UPF consumption and various other cancers and chronic diseases, global discussions around reducing overall UPF intake are still warranted. More extensive research is needed to investigate the potential role of UPF consumption in prostate cancer, utilizing more robust food classification systems and precise dietary assessments over longer periods of time to better understand any connections. Further studies using novel methodologies that can reliably capture the breadth and depth of data inclusive of long-term dietary intake are much needed and will help clarify whether UPFs or specific components within this broad category, play a role in prostate cancer development. This section is mandatory, with one or two paragraphs to end the main text.

## Figures and Tables

**Table 1:** Characteristics of the included studies.

Author, Year, Country	Period Conducted (Mean Follow-Up Time)	Sample Size, Prostate Cancer Cases	Study Design, Population	Dietary Assessment Method	Comparison of Dietary Exposure (UPF Consumption)	Outcome of Interest	Ascertainment of Prostate Cancer
Fiolet, 2018; France [14].	2009–2014 (5 years)	104,980; 281	Cohort; Adults >18 (NutriNet-Santé)	24-h dietary recall	Quartile 4 vs. Quartile 1	Risk of various cancers	Medical records
Kliemann, 2023; Multi-country * [25].	1991–2005 (14.1 years)	450,111; 6926	Cohort; Middle-aged adults (EPIC)	FFQ or country-specific dietary questionnaires	Quartile 4 vs. Quartile 1	Risk of various site-specific cancers	Cancer registries, cancer/pathology centers, health insurance records, active follow-up
Chang, 2023; UK [23].	2009–2019 (10 years)	197,426; 3261	Cohort; Adults 40–60 years (UK Biobank)	24-h dietary recall	Quartile 4 vs. Quartile 1	Risk of various site-specific cancers	National cancer and mortality registries
Trudeau, 2020; Canada [26].	2005–2012 (cases confirmed from 2005–2009)	3910; 1919 cases, 1991 controls	Case–control; Adults < 76 y/o (PROtEuS)	63-item FFQ	Quartile 4 vs. Quartile 1	Risk of prostate cancer	Histologically confirmed cancer cases via included hospitals
Romaguera, 2021; Spain [16].	2008–2013 (dietary information gathered 2.1 months after diagnosis on average)	7834; 953 cases, 1283 controls	Case–control; Adults 20–85 y/o (MCC-Spain)	140-item semiquantitative FFQ	Tertile 3 vs. Tertile 1	Risk of prostate, breast, and colorectal cancers	Histologically confirmed cancer cases via pathology departments
Pu, 2022; USA [24].	1993–2009 (10.76 years)	15,103; 4336	Cohort; Adults aged 55–74 y/o (PLCO Screening Trial)	124-item DHQ	Tertile 3 vs. Tertile 1	Cancer-related/all-cause mortality in prostate, lung, colorectal, and ovarian cancer	Screening exams + medical record abstraction

FFQ = food-frequency questionnaire; DHQ = diet history questionnaire. * = Denmark, France, Germany, Greece, Italy, the Netherlands, Norway, Spain, Sweden, and the UK.

**Table 2:** Main findings of included studies.

Author, Year, Country	Outcome of Interest	Covariates Adjusted *	Statistical Methods	Results	Conclusion
Fiolet, 2018; France [14].	Prostate cancer incidence	Education, Nutritional Quality of Diet (e.g., Lipid, Sodium, Carbohydrate).	Cox proportional hazards model	No association (Each 10% increase in UPF)	Higher UPF intake not linked to prostate cancer
Kliemann, 2023; Multi-country [25].	Prostate cancer incidence	Education, Nutritional Quality of Diet (e.g., Fiber, Fat, Sodium, Carbohydrates), Other Dietary Factors (Mediterranean Diet).	Cox proportional hazards model	No association (Quartile 4 vs. Quartile 1)	No significant association between UPF intake and cancer risk
Chang, 2023; UK [23].	Prostate cancer incidence	Ethnicity, Education, Nutritional Quality of Diet (e.g., Sodium, Total Fat, Carbohydrate, Fiber), Household Income.	Cox proportional hazard model	No association (Quartile 4 vs. Quartile 1)	Results aligned with NutriNet-Santé for prostate cancer incidence
Trudeau, 2020; Canada [26].	Prostate cancer risk	Education, Marital Status, Prostate Screening (PSA/DRE), Diabetes Status.	Logistic regression	OR 1.29 (processed foods), OR 0.86 (minimally processed)	Slight risk increase with processed foods; no UPF association
Romaguera, 2021; Spain [16].	Prostate cancer risk	Nutritional Quality of Diet (e.g., Fiber, Fatty Acids, Consumption of Fruits/Vegetables).	Logistic regression	No association (Tertile 3 vs. Tertile 1, OR 1.06)	No link between UPF intake and prostate cancer
Pu, 2022; USA [24].	Prostate cancer mortality	Race/Ethnic Group, Nutrient Intake (e.g., Fiber, Added Sugar, Fatty Acids), History of Diabetes, History of Hypertension, Food Groups and Servings (e.g., Vegetables, Fruit, Coffee, Red and Processed Meat).	Multivariable Cox regression	Significant association (Tertile 3 vs. Tertile 1, HR 1.15)	Higher UPF intake associated with increased prostate cancer risk

* listed are the covariates that do not overlap in all studies. Covariates in common across all studies include age, BMI, physical activity, smoking status, alcohol intake, energy intake, and history of cancer.

## Supplementary Materials

The following supporting information can be downloaded at https://www.mdpi.com/article/10.3390/cancers16233953/s1. Table S1: Assessment of study quality. Figure S1: Contour-Enhanced Funnel Plot for risk of development prostate cancer and prostate cancer mortality.
