# Supplementary material for: Ultra-Processed Food and Prostate Cancer Risk: A Systemic Review and Meta-Analysis
Source: Cancers (Basel). 2024 Nov 26;16(23):3953. doi: 10.3390/cancers16233953 (PMC11639853; doi:10.3390/cancers16233953)
Supplement: Supplementary file 1 [file cancers-16-03953-s001.zip › cancers-3296741-supplementary.pdf]

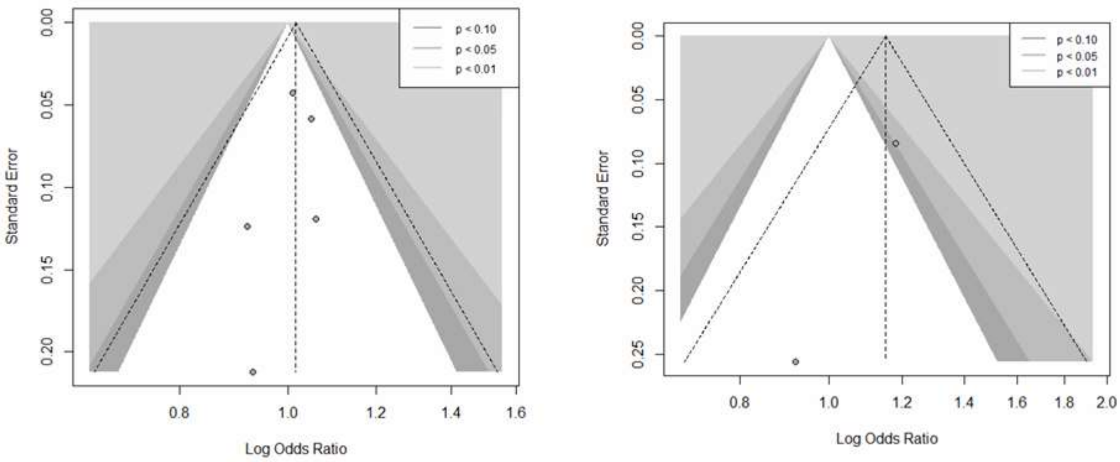

Supplement Figure S1 Contour-Enhanced Funnel Plot for risk of development prostate cancer and prostate cancer mortality.

Supplement Table S1

**Studies**	**Selection**	**Comparability**	**Outcome/Exposure**	**Score**
**Cohort**				
Fiolet 2018 [14].	3	2	3	8
Kliemann 2023 [25].	3	2	3	8
Chang 2023 [23]	3	2	3	8
Pu 2023 [24].	3	1	3	7
**Case Control**				
Trudeau 2020 [26].	4	2	3	9
Romaguera 2021 [16].	3	2	2	7
